# Improvements to a Markerless Allelic Exchange System for *Bacillus anthracis*

**DOI:** 10.1371/journal.pone.0142758

**Published:** 2015-12-01

**Authors:** Roger D. Plaut, Scott Stibitz

**Affiliations:** Division of Bacterial, Parasitic, and Allergenic Products, Center for Biologics Evaluation and Research, Food and Drug Administration, 10903 New Hampshire Ave., Silver Spring, Maryland, United States of America; ContraFect Corporation, UNITED STATES

## Abstract

A system was previously developed for conducting I-*Sce*I-mediated allelic exchange in *Bacillus anthracis*. In this system, recombinational loss of a chromosomally-integrated allelic exchange vector is stimulated by creation of a double-stranded break within the vector by the homing endonuclease I-*Sce*I. Although this system is reasonably efficient and represents an improvement in the tools available for allelic exchange in *B*. *anthracis*, researchers are nonetheless required to “pick and patch” colonies in order to identify candidate "exchangeants." In the present study, a number of improvements have been made to this system: 1) an improved I-*Sce*I-producing plasmid includes *oriT* so that both plasmids can now be introduced by conjugation, thus avoiding the need for preparing electro-competent cells of each integration intermediate; 2) antibiotic markers have been changed to allow the use of the system in select agent strains; and 3) both plasmids have been marked with fluorescent proteins, allowing the visualization of plasmid segregation on a plate and obviating the need for “picking and patching.” These modifications have made the process easier, faster, and more efficient, allowing for parallel construction of larger numbers of mutant strains. Using this improved system, the genes encoding the tripartite anthrax toxin were deleted singly and in combination from plasmid pXO1 of Sterne strain 34F2. In the course of this study, we determined that DNA transfer to *B*. *anthracis* could be accomplished by conjugation directly from a methylation-competent *E*. *coli* strain.

## Introduction

*Bacillus anthracis*, a spore-forming Gram-positive bacterium, is the causative agent of the disease anthrax, which can infect mammalian hosts by the respiratory, cutaneous, or gastrointestinal route. The genome of this organism consists of three genetic elements: the 5.2 Mb chromosome; plasmid pXO1, which includes genes encoding the tripartite anthrax toxin; and plasmid pXO2, which encodes the capsule [1]. Identification and characterization of genes encoding virulence factors and the detailed study of other aspects of gene function of any bacterial pathogen are facilitated by the ability to make desired modifications to such genetic elements.

Of all the types of tools available for the manipulation of bacterial genomes, those that can orchestrate and direct allelic exchange are arguably the most versatile. Insertions, deletions, and substitutions are possible and can be "seamless," i.e. leaving no "scar" or undesired sequences at novel DNA joints. Particularly with the advent of fast and affordable DNA synthesis, one is limited only by one's imagination. There is no theoretical limit to the size of alterations, although practical considerations, based on the types and efficiencies of modes of genetic transfer available for any given organism, may limit the size of introduced segments. Those comprising tens of kilobases are generally feasible. Importantly, allelic exchange can be "markerless," whereas most mutagenesis techniques, including transposon insertion, targetron-based mutagenesis, and some forms of recombineering, require the existence of a selectable marker, typically an antibiotic resistance gene. Subsequent removal of such a marker, to allow subsequent rounds of mutagenesis and to avoid concerns over the presence of antibiotic resistance genes, can be accomplished by the use of site-specific recombination sites flanking such a marker. However, these methods leave a DNA "scar" comprising, minimally, one copy of the recombination site. This can lead to complications in subsequent rounds of modification because of the ability of these sites to participate in chromosomal rearrangements. A recombineering approach using synthetic oligonucleotides can achieve efficiencies great enough to allow the incorporation of mutations without the use of a selectable marker. However, while the number of organisms in which recombineering can be used is growing, it is not currently available as a standard technique in many bacteria of interest, especially gram-positives. In addition, incorporation of unselected alterations is theoretically limited to those of relatively small size, capable of being encoded by an oligonucleotide that can be incorporated at DNA replication forks.

Allelic exchange results from, and requires, the occurrence of two homologous recombination events between the bacterial genome and an incoming DNA molecule. These occur within a segment of a construct that contains sequences homologous to the native genomic sequences, flanking the desired alteration. The first of these recombination events has typically been accomplished by the use of suicide vectors, or conditionally replicating vectors, typically plasmids. Such a plasmid vector can be maintained only after incorporation into the chromosome or other autonomously replicating genetic element. Selection for even rare events of this type can be accomplished, even at low efficiencies and with very low background, by virtue of an antibiotic resistance gene on the vector. Allelic exchange can be accomplished using a vector that is this simple, by subsequent screening for loss of the vector by homologous recombination. While such screening can be as rudimentary as replica plating or "picking and patching" of colonies, a more efficient approach incorporates some means of forcing, selecting for, or catalyzing loss of the plasmid vector. This can occur via a second homologous recombination event and leads to the incorporation of the desired alteration at frequencies theoretically approaching 50%. Approaches using a dominant streptomycin-sensitive *rpsL* gene [2] or the *sacB* gene of *Bacillus subtilis* [3] have been successful in a variety of gram-negative organisms.

A different approach to facilitating the second recombination event involves the use of site-specific cleavage, mediated by the restriction enzyme I-*Sce*I, of a cognate I-*Sce*I site present in the allelic exchange vector, but nowhere else in the bacterial chromosome. Induction of such cleavage leads to loss of the vector at high frequencies, presumably by both selecting against bacteria in which the chromosome has been damaged and by stimulating recombination of the flanking homologous sequences to repair the lesion. Originally reported for use in *Escherichia coli* [4], this approach was adapted for use in *B*. *anthracis* [5]. This system included a plasmid shuttle vector (pBKJ236), mobilizable from *E*. *coli* to *B*. *anthracis* by conjugation, which carried a temperature-sensitive origin of replication for conditional replication in Gram-positive bacteria and a target site for cleavage by the restriction enzyme I-*Sce*I. DNA carrying the desired modifications to the *B*. *anthracis* genome, along with flanking regions of homology, can be cloned into this vector. As a consequence of homologous recombination between plasmid and host sequences, the plasmid can be forced to integrate into the *B*. *anthracis* genome by growth at elevated temperatures (37°C) concomitant with selection for the erythromycin resistance marker on this vector. After the subsequent introduction of a second, "facilitator" plasmid expressing the I-*Sce*I restriction enzyme (pBKJ223), a double-stranded break within the integrated vector is induced. Repair of this break by a second recombination event results in loss of the integrated plasmid and either reversion to the wild-type allele or incorporation of the desired modification. This system has been used to make in-frame deletions in genetic elements of *B*. *anthracis* [6–15], *B*. *cereus* [16–18], *B*. *subtilis* [19, 20], and *B*. *thuringiensis* [21].

Although reasonably efficient, the procedure as described has several drawbacks. First, the antibiotic resistance genes encoded in the two plasmids (erythromycin and tetracycline) are not suitable for use in select agents. Second, in order to allow transfer of pBKJ223 into *B*. *anthracis* by transformation, electrocompetent cells must be prepared. Third, screening for the loss of plasmid DNA still requires some “picking and patching" to identify antibiotic-sensitive segregants. The goal of the present work was to overcome these issues, making the whole procedure easier, faster, and more efficient.

## Materials and Methods

### Bacterial strains

*Bacillus anthracis* strains 7702 and 34F2 were grown on brain heart infusion (BHI) agar or in BHI broth, supplemented with spectinomycin (250 μg/ml) or kanamycin (20 μg/ml) as necessary. When required, BHI agar was also supplemented with polymyxin B (60 units/ml) to inhibit growth of gram-negative bacteria. *E*. *coli* strain DH5alpha was used for cloning experiments. In some experiments, strains SCS110, SM10, and S17.1 were used for transfer of plasmids into *B*. *anthracis*; in other experiments, strain SS1827 [22] was used as a helper for conjugation. *E*. *coli* strains were grown on LB agar or in LB broth, supplemented with spectinomycin (100 μg/ml), kanamycin (25 μg/ml), or ampicillin (100 μg/ml) as necessary.

### Plasmids

**Fig 1:**
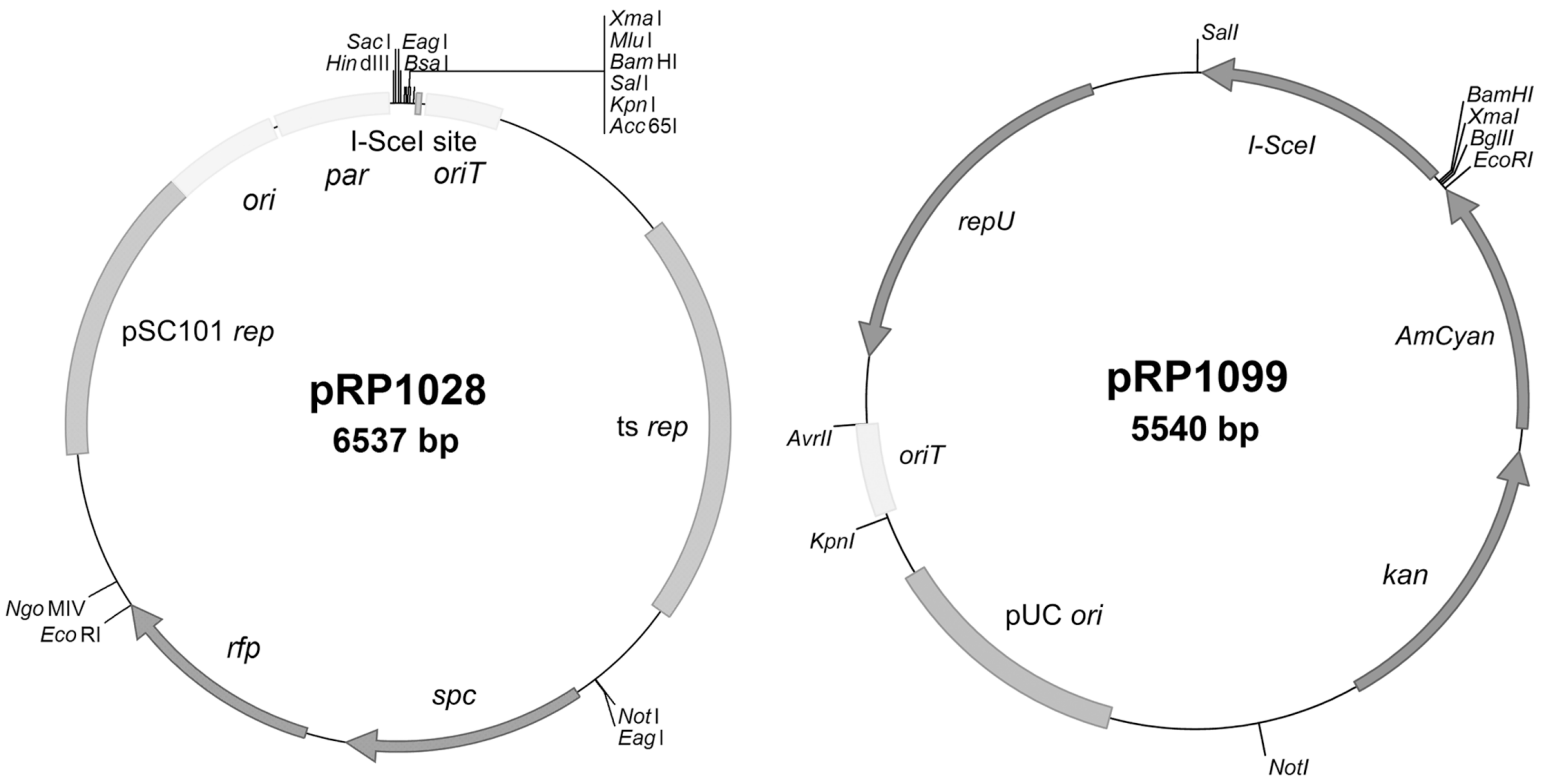
Improved plasmids for allelic exchange. See Materials and Methods for descriptions.

Detailed methods regarding the development of the improved plasmids are in S1 Methods. Most features of shuttle vector pRP1028 (GenBank accession number KT823412) were derived from the same sources as those of pBKJ236 [5], including the pSC101 replicon for replication in *E*. *coli* [23], a temperature-sensitive replicon from pWV01 for conditional replication in *Bacillus* [24], *oriT* of RP4 [25], and an I-*Sce*I site [26]. Differences between pRP1028 and pBKJ236 are as follows: The erythromycin resistance gene was replaced by the spectinomycin adenyltransferase gene *aad9* from *Staphylococcus aureus* transposon Tn554 [27] under the control of the *trc* promoter [28]; the sequence encoding the fluorescent protein TurboRFP [29] was codon-optimized for *B*. *anthracis*, synthesized (GenScript, Piscataway, NJ), and inserted downstream of the *B*. *subtilis rrnB* P2 promoter [30] and the promoter PFP2 (TTGACAGTATAAAGTTAGAAACTTATAAT); and the multiple cloning site of pBKJ236 was modified for convenience. The resulting shuttle vector was designated pRP1028 (Fig 1).

Facilitator plasmid pRP1099 (GenBank accession number KT823414) was engineered with many of the elements of pBKJ223 [5], with the following differences: The pSC101 replicon for replication in *E*. *coli* was replaced with a pUC replicon [31]; the promoter controlling the gene for I-*Sce*I was removed, although the ribosome binding site of *pagA* was retained; to permit transfer from *E*. *coli* to *B*. *anthracis* by conjugation, *oriT* of RP4 [25] was added; tetracycline resistance was replaced by the kanamycin resistance gene *aphA(3)* from *Staphylococcus aureus* [32], under the control of its native promoter; and the gene for the fluorescent protein AmCyan (BD Biosciences, San Jose, CA) was added under the control of the promoter PFP1 (TTGATAGTATAAAGTTAGAAACTTATAAT). The resulting facilitator plasmid was designated pSS4332 (GenBank accession number KT823413). Subsequently, pSS4332 was found to contain tandem copies of both *oriT* and the gene for AmCyan. These duplications were unintended and were not recognized initially. The repeats were removed, and the modified plasmid was designated pRP1099 (Fig 1). Results obtained using pRP1099 were comparable to those obtained using pSS4332 (data not shown).

Deletion constructs were engineered by cloning homology to pXO1 into pRP1028. Primers are listed in S1 Table. Briefly, for deletion of *cya*, plasmid pBKJ244 [33] was digested with NotI, and the resulting 917-bp insert fragment was cloned into pRP1028 that had been digested with BsaI, generating pRP1110. For deletion of *lef*, primers 212 and 213 were used to amplify sequence that included the *Δlef243* allele from BA723 [33]. The resulting PCR product was digested with BsaI and cloned into pRP1028 that had been digested with BamHI and BsaI, generating pRP1091. The same strategy was used for deletion of *pagA*, using primers 214 and 215 with strain BA690 [33] as template, generating pRP1101. Sequencing confirmed the presence of the desired DNA inserts.

### Transformation and conjugation

Transfer of plasmids from *E*. *coli* to *B*. *anthracis* by electrotransformation was performed as described previously [5]; plasmids were first transformed into the *E*. *coli dam dcm* strain SCS110 (Stratagene, La Jolla, CA). For transfer of plasmids by conjugation, a procedure described previously [5] was modified as follows: For transfer of pRP1028 or derivatives to *B*. *anthracis* by triparental conjugation, agar plates containing the appropriate antibiotics were streaked with SS1827, the *B*. *anthracis* recipient strain, and the *E*. *coli* strain harboring pRP1028 or derivative; plates were incubated overnight at 37°C. Overnight growth of each strain was collected with a sterile polyester swab and transferred to a single BHI plate. The three strains were mixed together on the plate for 60–90s, and the plate was incubated for at least 24h at room temperature (approximately 25°C). Growth was transferred by swab to BHI plates containing spectinomycin and polymyxin B and incubated 2d at room temperature. The same procedure was used for transfer of pRP1099 into *B*. *anthracis* by triparental mating, except that the mating plate was incubated 6–8h at 37°C, after which growth was transferred to a BHI plate containing kanamycin and polymyxin B and incubated overnight at 37°C.

For biparental matings, plasmids were transferred to *E*.*coli* strains expressing the conjugation apparatus before mating with the *B*. *anthracis* recipient strain; pRP1028 derivatives were transformed into SM10 (Km^r^) whereas pRP1099 was transformed into S17.1 (Spc^r^) [34].

### Imaging

An LAS-3000 imager (Fuji Medical Systems, Stamford, CT) was used for visualization of fluorescent bacteria. Fluorescence of bacterial strains carrying pRP1028 and derivatives (TurboRFP) was assessed under green light (wavelength of 520 nm) using a 605DF40 emission filter, whereas fluorescence of strains carrying pRP1099 (AmCyan) was assessed under blue light (460 nm) using a 510DF10 emission filter. Typical exposure settings were 1–10s at an aperture of F2.8.

## Results and Discussion

The improved plasmids are depicted in Fig 1. Shuttle vector pRP1028 includes a gene for TurboRFP [29], codon-optimized for *B*. *anthracis*. Fluorescence of strains harboring pRP1028 or derivatives can be visualized under green light (wavelength of 520 nm). Similarly, facilitator plasmid pRP1099 includes a gene for AmCyan (BD Biosciences, San Jose, CA), enabling the fluorescence of strains harboring the plasmid to be visualized under blue light (wavelength of 460 nm).

The procedure for allelic exchange is outlined in Table 1. Conjugation of *B*. *anthracis* with pRP1028 derivatives (carrying deletion or other mutation constructs flanked by homologous sequences) is conducted at room temperature (approximately 25°C) and allowed to proceed for 24h. Conjugations can be biparental or triparental (see Materials and Methods). The mating mixture is then transferred to BHI containing spectinomycin (to select for retention of pRP1028) and polymyxin B (to inhibit growth of the *E*. *coli* donor and/or helper strain) and incubated 2d at room temperature. Growth is then visualized under conditions for TurboRFP (see Materials and Methods). Fluorescent colonies of *B*. *anthracis* are passed, and subsequent incubations are conducted at the non-permissive temperature for plasmid replication (37°C). Growth is visualized again under the same conditions, and fluorescent colonies (in which the shuttle plasmid has integrated into *B*. *anthracis* genomic DNA following crossover by homologous recombination) are passed three times. In a second round of conjugation, pRP1099 is transferred to the integrant, to stimulate the second crossover by expression of I-*Sce*I and cleavage of the integrated plasmid. Colonies are pooled and streaked on BHI containing kanamycin. Re-imaging for TurboRFP expression allows visualization of colonies that have lost the integrated pRP1028 derivative following a second crossover event. Non-fluorescent strains are screened by PCR for incorporation of the desired alteration to the *B*. *anthracis* genome. In a final step, strains with the desired change are passed in the absence of antibiotics and visualized under conditions for AmCyan to identify colonies that have lost pRP1099.

**Table 1:** Allelic exchange procedure.

Day	Procedure
D0	Streak donor, recipient, and helper strains; incubate overnight at 37°C.
D1	Conjugation of donor, recipient, and helper on BHI; incubate 24h at room temperature (RT).
D2	Swab growth and transfer to BHI + 250 μg/ml spectinomycin + 60 U/ml polymyxin B (BHI-SpcPmx); streak for isolation and incubate 2d at RT.
D4	View plate under green light; pick a fluorescent colony, pass to BHI-SpcPmx, and incubate overnight at 37°C.
D5	Repeat D4.
D6	Repeat D4.
D7	Repeat D4; streak RP1099 and helper strain; incubate overnight at 37°C.
D8	Conjugation of integrant, RP1099, and helper strain on BHI; incubate 6–8h at 37°C; swab growth and transfer to BHI + 20 μg/ml kanamycin + 60 U/ml polymyxin B (BHI-KanPmx); streak for isolation and incubate overnight at 37°C.
D9	Pool 10–20 colonies, streak on BHI-KanPmx, and incubate overnight at 37°C.
D10	View plate under green light; pick non-fluorescent colonies, patch on BHI-SpcPmx and BHI; incubate overnight at 37°C.
D11	PCR-screen Spc-sensitive strains; pass desired strain(s) onto BHI and incubate overnight at 37°C.
D12	View plate under blue light; pick non-fluorescent colonies, patch on BHI-Kan and streak on BHI.
D13	Make a freezer stock of a Kan-sensitive strain.

**Fig 2:**
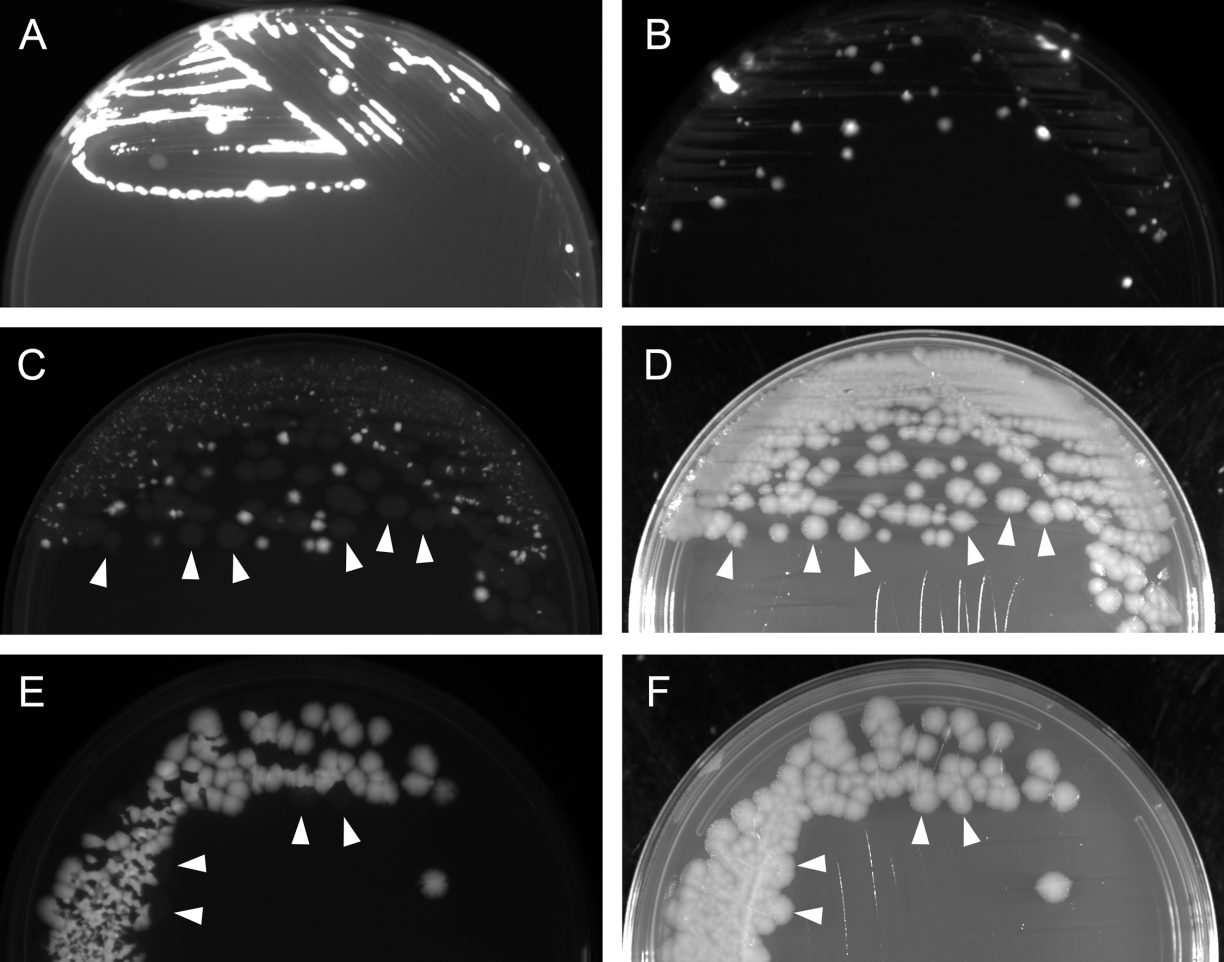
Utility of fluorescent proteins. Representative images obtained during the procedure (outlined in Table 1) of engineering strain BAP325 (Sterne 34F2 *Δlef*). Agar plates were imaged with a Fuji LAS-3000 imager. A: pRP1091 in Sterne strain 34F2, imaged under green light (520 nm) on D4 of the procedure, after incubation for 2d at RT. Fluorescent *B*. *anthracis* (large, circular colonies) indicates presence of freely replicating pRP1091. B: pRP1091 in 34F2, imaged under green light on D5, after incubation overnight at 37°C. Fluorescence indicates integration of pRP1091 into pXO1. C, D: Images obtained on D10, after transfer of pRP1099 to the integrant via conjugation, under green light (C) and white light (D). Lack of fluorescence (arrowheads) indicates loss of the integrated pRP1091 from pXO1. E, F: Images obtained on D12, after passage in the absence of kanamycin, under blue light (460 nm; E) and white light (F). Lack of fluorescence (arrowheads) indicates loss of pRP1099.

**Fig 3:**
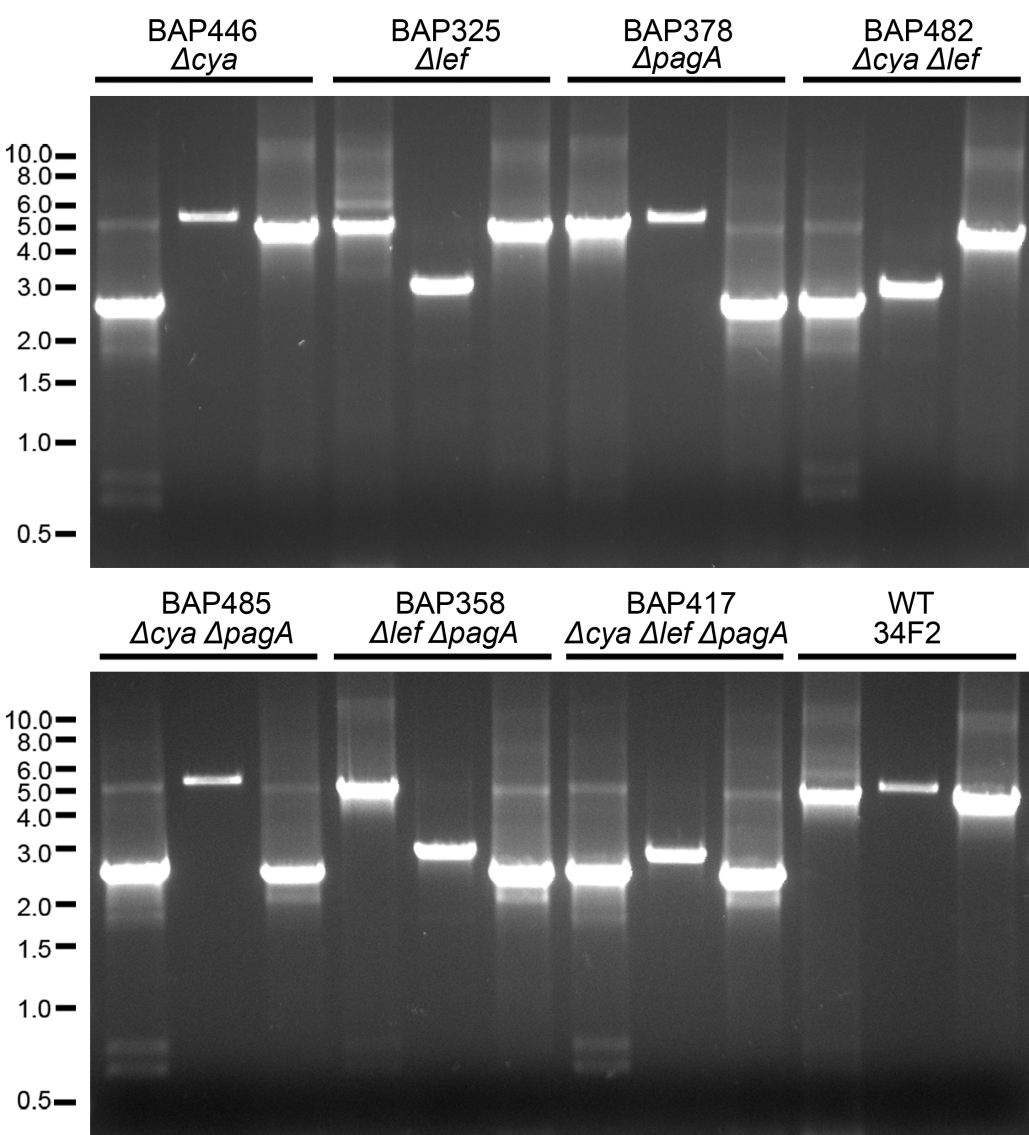
Diagnostic PCR of mutant strains. Strains are listed in Table 3. PCR products were run on a 0.8% agarose gel with ethidium bromide. For each strain, the three lanes represent PCR products using primer pairs to amplify, from left to right, the regions of *cya*, *lef*, and *pagA* on pXO1, with primers listed in S1 Table. Ladder indicates size in kb.

To demonstrate the use of the improved plasmid system, we undertook deletion of the genes encoding the tripartite anthrax toxin, singly and in combination, from pXO1 of the Sterne strain 34F2. Deletion constructs, including 450–1000bp of flanking homology, were cloned into pRP1028 (Table 2). After the conjugation procedure and incubation for 2d at room temperature, growth was observed under green light (Fig 2A), and fluorescent colonies were picked and passed three times at 37°C. Subsequent fluorescence of growth under green light (Fig 2B) suggested that the pRP1028 derivative had integrated into pXO1. Following conjugation with pRP1099, loss of the integrated plasmid was detected by a third round of visualization under green light (Fig 2C and 2D). PCR screening of such colonies led to the isolation of strains in which *cya*, *lef*, and/or *pagA* were deleted. Finally, strains no longer carrying pRP1099 could be isolated by observing colonies under blue light (Fig 2E and 2F). PCR results confirmed that one, two, or all three of the toxin genes were deleted (Fig 3).

**Table 2:** Description of plasmid constructs.

Allele	Plasmid	Upstream homology (bp)	Mutation	Downstream homology (bp)	Size of deletion (bp)
*Δcya*	pRP1110	447	ATG-CAATTG-TAA	450	2397
*Δlef*	pRP1091	509	ATG-CAATTG-TAA	492	2424
*ΔpagA*	pRP1101	1078	ATG-11 codons-GAATTC-3 codons-TAA	1011	2247

**Table 3:** Description of deletion mutant strains.

Allele(s)	Strain
*Δcya*	BAP446
*Δlef*	BAP325
*ΔpagA*	BAP378
*Δcya Δlef*	BAP482
*Δcya ΔpagA*	BAP485
*Δlef ΔpagA*	BAP358
*Δcya Δlef ΔpagA*	BAP417

In our laboratory, this improved system has been used successfully to engineer point mutations, insertions, and deletions in the chromosome and plasmid pXO1 of Sterne strains 34F2 and 7702 (data not shown).

Previous studies have shown that efficient transfer of DNA from *E*. *coli* to *B*. *anthracis* by transformation requires passage of plasmids through a methylation-deficient strain such as SCS110 [35]. During the course of this study, we found that the efficiency of transfer of pRP1028 from DH5α to *B*. *anthracis* by conjugation was comparable to that of conjugation using SCS110 (data not shown). During electrotransformation, double-stranded plasmid DNA is taken up by the recipient strain, whereas during conjugation, a single strand is transferred, and subsequent synthesis of the complementary strand occurs in the recipient cell [36, 37]. We speculate that bi-methylated DNA from DH5α (which would be transferred to the recipient during transformation) is a substrate for restriction by *B*. *anthracis* endonuclease(s). However, conjugation proceeds by transfer of a single strand, with the complementary strand being synthesized in the recipient cell. This should result in hemi-methylated DNA, which we hypothesize is not a substrate for *B*. *anthracis*-encoded restriction endonucleases.

During the course of our work with this improved system, when we transferred DNA that had been passed through the *dam dcm* strain SCS110 to *B*. *anthracis* by electrotransformation, we subsequently isolated strains that carried integrated pRP1028 derivatives but did not exhibit red fluorescence. Analysis of these strains showed that the coding sequence for TurboRFP was interrupted by the insertion of IS10 (data not shown). Our interpretation of these results is that high expression of TurboRFP is disadvantageous for *E*. *coli* and that selection occurs for strains with IS10 insertions. IS10 is present in some genetically modified strains of *E*. *coli* because of the use of Tn10 during their development [38]. SCS110 is a derivative of JM110, which was engineered using Tn10 [31]. Moreover, IS10 transposition is increased in *dam* strains [39]. However, IS10 is not active in DH5α, according one report [40], and it is not predicted to be present in SM10 [34, 39]. Therefore, rather than transferring plasmid DNA to *B*. *anthracis* by performing electrotransformation of *B*. *anthracis* using DNA isolated from SCS110, we now routinely perform conjugations using DH5α or SM10 as the donor strain. This approach has eliminated the problem of non-fluorescent integrants.

The improvements described here to the system for performing allelic exchange render it suitable for use in select agent strains. Additionally, the ability to transfer both plasmids into *B*. *anthracis* by conjugation and the inclusion of fluorescent protein markers make the procedure less time-consuming, facilitating its use for performing multiple experiments in parallel and for engineering strains with multiple point mutations, insertions, or deletions.

## Supporting Information

S1 Methods Plasmid construction. (PDF)

S1 Table Oligonucleotides used in this work. (PDF)
