# Supplementary material for: Improvements to a Markerless Allelic Exchange System for Bacillus anthracis
Source: PLoS One. 2015 Dec 1;10(12):e0142758. doi: 10.1371/journal.pone.0142758 (PMC4666636; doi:10.1371/journal.pone.0142758)
Supplement: S1 Methods — (PDF) [file pone.0142758.s001.pdf]

S1 Methods. Plasmid construction

Plasmid pRP1028 was constructed from pBKJ236 [1] as follows: To remove the EcoRI site near *oriT*, a kanamycin resistance cassette was amplified from pKANπ [2] using primers SS965 and SS966. The resulting PCR product was digested with MfeI and inserted into pBKJ236 that had been partially digested with EcoRI, yielding plasmid pSS4004. This plasmid was then digested with MluI and self-ligated, removing the kanamycin resistance cassette and the EcoRI site, yielding plasmid pSS4005. The same approach was used to insert the kanamycin resistance gene at two additional EcoRI sites in pSS4005 in parallel, using primers SS967 and SS968 each time and yielding plasmids pSS4006 (KanR at the EcoRI site near *lac* promoter) and pSS4007 (KanR at the EcoRI site near 3' end of *ery*). To remove the *lac* promoter and the nearby EcoRI site, pSS4006 was digested with EcoRI and BglII, and the small fragment was joined with the large fragment that resulted from digestion of pSS4007 with EcoRI and BamHI, yielding plasmid pSS4008. To add a NotI site, the kanamycin resistance cassette was amplified using primers 969 and 970, and the resulting PCR product was digested with EcoRI and inserted into pSS4008 at the EcoRI site, yielding plasmid pSS4028; this plasmid was then digested with NotI, and the two large fragments were retained and ligated together, yielding plasmid pSS4030. To add back *oriT*, primers SS1897 and SS1904 were used in a reaction with plasmid D2BpUC9Cam [3] as template. The resulting product was digested with BsaI and PstI and was inserted into pSS4030 that had been digested with NsiI and Acc65I, yielding plasmid pU. To add a multiple cloning site, overlapping oligonucleotides SS1858 and SS1859 were combined in a ligation mixture with HindIII-KpnI-digested pU, yielding plasmid pV. Codon-optimized TurboRFP [4] synthesized by GenScript (Piscataway, NJ) under the control of a strong constitutive promoter, PFP2 (TTGACAGTATAAAGTTAGAAACTTATAAT), was amplified using primers RP41 and RP42, and the PCR product was digested with EcoRI and BamHI. Additionally, the spectinomycin resistance gene *aad9*, originally from Tn554 [5] was amplified using primers RP39 and RP40, and the PCR product was digested with NotI and BamHI. The two digested PCR

products were combined in a three-fragment ligation mixture with NotI-EcoRI-digested pV, and the resulting plasmid was designated pRP1018. To insert an additional promoter (the *B. subtilis rrnB* P2 promoter [6]), pRP1018 was digested with BsaI and combined in a ligation mixture with the overlapping oligonucleotides RP61 and SS2065; the resulting plasmid was designated pRP1028.

Plasmid pRP1099 was engineered as follows: Inverse PCR was performed using pBluescript (Agilent Technologies, Santa Clara, CA) as template, with primers SS1338 and SS1329. The resulting PCR product was digested with BsaI and self-ligated, removing the BsaI site and resulting in plasmid pSS4176. To add resistance to kanamycin, the cassette was PCR-amplified from pTN1 [7] using primers SS1525 and 1555. The PCR product was digested with BamHI and NotI and cloned into pSS4176 at the same sites, yielding plasmid pSS4176. The fluorescent protein AmCyan was amplified from pAmCyan (BD Biosciences, San Jose, CA) using primers SS1559 and SS1564. The PCR product was digested with EcoRI and BamHI and inserted into pSS4176 that had been digested with the same enzymes, yielding pSS4375. Promoter PFP1 (<u>TTGATA</u>GTATAAAGTTAGAAACT<u>TATAAT</u>) was inserted upstream of AmCyan by digesting pSS4375 with BsaI and combining the digested plasmid in a ligation mixture with the overlapping oligonucleotides SS1593 and SS1594, yielding pSS4386. To remove the ampicillin resistance cassette and other extraneous DNA sequences, the pUC replicon was amplified from pSS4386 using primers SS1575 and SS1576. The resulting PCR product was digested with KpnI and NotI and was cloned into the smaller fragment that resulted from digestion of pSS4386 with the same enzymes, yielding plasmid pW. The Gram-positive replicon, including *repU*, originally from pBC16 [8], was amplified from pBKJ223 [9] using primers SS1577 and SS1578. The PCR product was digested with KpnI and inserted into KpnI-digested pW, yielding plasmid pX. To remove undesirable restriction sites, plasmid pX was digested with SalI and self-ligated, yielding plasmid pY. To add *oriT*, primers SS1454 and SS1455 were used in a PCR reaction with pBKJ236 as template. The resulting PCR product was digested with KpnI and was cloned into KpnI-digested pY, resulting in plasmid pZ. The I-*Sce*I gene was amplified from pBKJ223 using primers SS1244 and SS1245. The PCR product was digested with EcoRV

and SalI and cloned into pZ that was partially digested with SalI and EcoRV, yielding pSS4332. During the construction of pSS4332, tandem duplications of both *oriT* and the gene for AmCyan were inadvertently inserted. To remove the *oriT* tandem repeats, pSS4332 was digested with AvrII and KpnI. Primers SS1455 and RP211 were used to amplify *oriT* from pRP1028. The resulting PCR product was digested with AvrII and KpnI and cloned into pSS4332 at the same sites, yielding plasmid pRP1092. To remove the tandem duplication of the gene for AmCyan, pRP1092 was digested with EcoRI and self-ligated. Sequencing confirmed the presence of only one copy of AmCyan, and the resulting plasmid was designated pRP1099.

References

1. Janes BK, Stibitz S. Routine markerless gene replacement in Bacillus anthracis. Infection and immunity. 2006;74(3):1949-53. PubMed PMID: 16495572.

2. Black WJ, Falkow S. Construction and characterization of Bordetella pertussis toxin mutants. Infect Immun. 1987;55(10):2465-70. PubMed PMID: 2888733; PubMed Central PMCID: PMC260731.

3. Waters VL, Hirata KH, Pansegrau W, Lanka E, Guiney DG. Sequence identity in the nick regions of IncP plasmid transfer origins and T-DNA borders of Agrobacterium Ti plasmids. Proc Natl Acad Sci U S A. 1991;88(4):1456-60. PubMed PMID: 1996345; PubMed Central PMCID: PMC51037.

4. Merzlyak EM, Goedhart J, Shcherbo D, Bulina ME, Shcheglov AS, Fradkov AF, et al. Bright monomeric red fluorescent protein with an extended fluorescence lifetime. Nat Methods. 2007;4(7):555-7. PubMed PMID: 17572680.

5. Murphy E. Nucleotide sequence of a spectinomycin adenyltransferase AAD(9) determinant from Staphylococcus aureus and its relationship to AAD(3") (9). Mol Gen Genet. 1985;200(1):33-9. PubMed PMID: 2993813.

6. Deneer HG, Spiegelman GB. Bacillus subtilis rRNA promoters are growth rate regulated in Escherichia coli. J Bacteriol. 1987;169(3):995-1002. PubMed PMID: 3029043.

7. Le Breton Y, Mohapatra NP, Haldenwang WG. In Vivo Random Mutagenesis of Bacillus subtilis by Use of TnYLB-1, a mariner-Based Transposon. Appl Environ Microbiol. 2006;72(1):327-33. doi: 10.1128/aem.72.1.327-333.2006.

8. Bernhard K, Schrempf H, Goebel W. Bacteriocin and antibiotic resistance plasmids in Bacillus cereus and Bacillus subtilis. J Bacteriol. 1978;133(2):897-903. Epub 1978/02/01. PubMed PMID: 415051; PubMed Central PMCID: PMC222102.

9. Janes BK, Stibitz S. Routine Markerless Gene Replacement in Bacillus anthracis. Infect Immun. 2006;74(3):1949-53. doi: 10.1128/iai.74.3.1949-1953.2006.
