# Supplementary material for: Improvements to a Markerless Allelic Exchange System for Bacillus anthracis
Source: PLoS One. 2015 Dec 1;10(12):e0142758. doi: 10.1371/journal.pone.0142758 (PMC4666636; doi:10.1371/journal.pone.0142758)
Supplement: S1 Table — (PDF) [file pone.0142758.s002.pdf]

Supplemental Table 1. Oligonucleotides used in this work

Name	Sequence[a]	Use	Restriction site(s)
RP212	TAT GGTCTC GGATCC ATTGGCGATCATTAGTAGATCCTGG	Δ*pagA*	BsaI, BamHI
RP213	TAT GGTCTC CGGCCG AAACGCATAGGATGTGCCATTG	Δ*pagA*	BsaI, EagI
RP214	TAT GGTCTC GGATCC TTTGGCTTTAACGAAATGTATGTGC	Δ*lef*	BsaI, BamHI
RP215	TAT GGTCTC CGGCCG TTTCAGTTATTCATTCTGGATAGTC	Δ*lef*	BsaI, EagI
SS2164	CACGAGAAGAGTATTTAAAGAAAATC	diagnose Δ*lef*	none
SS2165	AACTATAGGACAATATTCATTACCATG	diagnose Δ*lef*	none
SS2166	ATATCAAGTTTAATTGTTAAGTTTGAAGG	diagnose Δ*cya*	none
SS2167	CCCGCGGCCGCAACCAAATGGTTTTCATTTCTTAG.	diagnose Δ*cya*	none
SS2168	CGCATATAAGCAAATACTTAATTGGTC	diagnose Δ*pagA*	none
SS2169	GGATAGGGTTTAACAACTTAATAATCCC	diagnose Δ*pagA*	none
SS965	CGC CAATTG ACGCGTCCACGTTGTGTCTCAAAATCTCTGA	amplify *kanR*	MfeI, MluI
SS966	CGC CAATTG ACGCGTCATCGCCCCATCATCCAGCCAGAAAG	amplify *kanR*	MfeI, MluI
SS967	CGC CAATTG ATGCAT AGATCT CCACGTTGTGTCTCAAAATCTCTGA	amplify *kanR*	MfeI, BglII
SS968	CGC CAATTG ATGCAT AGATCT CATCGCCCCATCATCCAGCCAGAAAG	amplify *kanR*	MfeI, BglII
SS969	CGC GAATTC CGTCTCAGAAAC GCGGCCGC CCACGTTGTGTCTCAAAATCTCTGA	amplify *kanR*	EcoRI, NotI
SS970	CGC GAATTC CGTCTCAGAAAC GCGGCCGC CATCGCCCCATCATCCAGCCAGAAAG	amplify *kanR*	EcoRI, NotI
SS1897	CGC GGTCTC CGTACGCGGCCAGCCTCGCAGAGCAGGATT	amplify *oriT*	BsaI
SS1904	CGC GGTCTC CTGCAG AAGCTTGGATCCGGATCGCGCTTTTCCGCTGCATA	amplify *oriT*	BsaI, PstI
SS1858	AGCTTGAGCTCCTAGGCGGCCGGAGACCTGTACACCCGGGACGCGTGGATCCGTCGACATGCATGGTACCAATTACCCTGTTATCCCTATAGTAC	add MCS	multiple
SS1859	TATAGGGATAACAGGGTAATTGGTACCATGCATGTCGACGGATCCACGCGTCCCGGGTGTACAGGTCTCCGGCCGCCTAGGAGCTCA	add MCS	multiple
RP39	GTCTCAGAAAC GCGGCCGC AGC	amplify *spcR*	NotI
RP40	ATA GGATCC GGTCTC GCATCAATTGAGAGAAGTTTCTATAGAA	amplify *spcR*	BamHI, BsaI
RP41	ATA GGATCC GGTCTC CGATGTTTGTTGACAGTATAAAGTTAGAAAC	amplify *turboRFP*	BamHI, BsaI
RP42	CGC GAATTC TTATCTATGCCCTAATTTACTAGGTAAATCAC	amplify *turboRFP*	EcoRI
SS2065	GATGGCAAAACAACTTGAAAAAAGTTGTTGACAAAAAAGAAGCTGAATGTTATATTAGTAAAGTCTC	add *rrnB* promoter	none
RP61	CATCGAGACTTTACTAATATAACATTCAGCTTCTTTTTTGTCAACAACTTTTTTCAAGTTGTTTTGC	add *rrnB* promoter	none
SS1328	CGC GGTCTC GCTCTCGCGGTATCATTGCAGCACT	amplify pBluescript	BsaI
SS1329	CGC GGTCTC GAGAGCCACGCTCACCGGCTCCAGA	amplify pBluescript	BsaI
SS1525	CGC GCGGCCGC GCCACATAGATGGCGTCGCT	amplify *kanR*	NotI
SS1555	CGC GGATCC GGTCTCTCATCAAAACAATTCATCCAGTA	amplify *kanR*	BamHI, BsaI
SS1559	CGC GAATTC TCAGAAAGGGACAACAGAGGTTATATGTGCAA	amplify a*mCyan*	EcoRI
SS1564	CGC GGATCC GGTCTC TGATGGCTCTTTCAAACAAGTTTATCGGA	amplify a*mCyan*	BamHI, BsaI
SS1593	GATGTTTGTTGATAGTATAAAGTTAGAAACTTATAATGATAAGTTTAATTGGAGGGAATTAT	add promoter PFP1	none
SS1594	CATCATAATTCCCTCCAATTAAACTTATCATTATAAGTTTCTAACTTTATACTATCAACAAA	add promoter PFP1	none
SS1575	CGC GCGGCCGC CAGCTTTTGTTCCCTTTAGTGAGGGTTAATTGC	amplify pUC *oriV*	NotI
SS1576	CGC GGTACC CTGATTAAGCATTGGTAACTGTCAGACCAAG	amplify pUC *oriV*	KpnI
SS1577	CGC GGTACC TAGGAATAATGAGGGCAGACGTAGTTTATAGGG	amplify pBC16 *oriV*	KpnI
SS1578	CGC GGTACC GTCGACAAAAGAACGAAGTCGAGATCAGGGAATGAG	amplify pBC16 *oriV*	KpnI
SS1454	CGC GGTACC CGGCCAGCCTCGCAGAGCAGGATT	amplify *oriT*	KpnI
SS1455	CGC GGTACC GGATCGCGCTTTTCCGCTGCATA	amplify *oriT*	KpnI
SS1244	GCG GTCGAC TCACTATTATTTCAGGAAAGTTTCGGAGGAGATAGTGT	amplify I-*Sce* I	SalI
SS1245	GCG GATATC GAGATCTCCCGGGGGATCCAATGAAAGGAGAACGCATATGCATCA	amplify I-*Sce* I	EcoRV
RP211	TAT CCTAGG CGGCCAGCCTCGCAGAGCAGGATT	amplify *oriT*	AvrII

[a]Restriction sites are underlined
